# Repeatability of intraocular pressure measurements with Icare PRO rebound, Tono-Pen AVIA, and Goldmann tonometers in sitting and reclining positions

**DOI:** 10.1186/1471-2415-13-44

**Published:** 2013-09-05

**Authors:** Caterina Schweier, James VM Hanson, Jens Funk, Marc Töteberg-Harms

**Affiliations:** 1 Department of Ophthalmology, UniversityHospital Zurich, Frauenklinikstrasse 24, 8091 Zurich, Switzerland; 2 Massachusetts Eye & Ear Infirmary, Harvard Medical School, 243 Charles Street, Boston, Massachusetts 02144, USA

## Abstract

**Background:**

Icare PRO (ICP) is a new Rebound tonometer that is able to measure intraocular pressure (IOP) in both sitting and reclining positions. In this study, the gold standard Goldmann tonometer (GAT) was compared to ICP and Tono-Pen AVIA (TPA). Hypothesis was that repeatability of GAT is superior to ICP and TPA.

**Methods:**

36 eyes of 36 healthy caucasian individuals, 13 male and 26 females, 17 right and 19 left eyes have been included in this prospective, randomized, cross-sectional study. The study was conducted at a single site (Dept. of Ophthalmology, UniversityHospital Zurich, Switzerland). Primary outcome measures were Intraclass correlation coefficients (ICC) and coefficients of variation (COV) and test-retest repeatability as visualized by Bland-Altman analysis. Secondary outcome measures were IOP in sitting (GAT, ICP and TPA) and in reclining (ICP and TPA) position.

**Results:**

Mean IOP measured by GAT was 14.9±3.5 mmHg. Mean IOP measured by ICP was 15.6±3.1 mmHg (with TPA 14.8±2.7 mmHg) in sitting and 16.5±3.5 mmHg (with TPA 17.0±3.0 mmHg) in reclining positions. COVs ranged from 2.9% (GAT) to 6.9% (ICP reclining) and ICCs from 0.819 (ICP reclining) to 0.972 (GAT).

**Conclusions:**

Repeatability is good with all three devices. GAT has higher repeatability compared to the two tested hand-held devices with lowest COVs and highest ICCs. IOP was higher in the reclining compared to the sitting position.

**Trial registration:**

The study was registered to the Clinical Trials Register of the US National Institute of Health, NCT01325324.

## Background

A precise measure of intraocular pressure (IOP) is essential in diagnosing and managing many ophthalmological diseases, particularly in glaucoma.

The gold standard in measuring IOP remains Goldmann applanation tonometry (GAT; Haag-Streit AG, Könitz, Switzerland) which was first introduced in 1957 by Hans Goldmann [1]. However, it is well known that corneal biomechanical properties influence the measured IOP value. For example, there is evidence that central corneal thickness (CCT) affects IOP readings by GAT in that IOP is underestimated in thin and overestimated in thick corneas [2-4]. Corneal edema [5], corneal astigmatism [6], refractive corneal surgery [7] and corneal hysteresis [8] also affect IOP readings.

For IOP measurement with GAT the patient must be able to sit at a slit lamp with a slit lamp-mounted GAT in an upright position. In some cases this is impossible, e.g. bed-ridden persons, small children, intraoperative assessments, and in other situations outside of the consulting room. In these cases hand-held tonometers are used. Many hand-held devices are available to measure IOP when the patient is either sitting upright or reclining horizontally. All have advantages and disadvantages compared to GAT. The Tono-Pen AVIA (TPA; Reichert Inc., Depew, New York, USA) is currently widely used. A more recently available option is the Icare PRO Rebound Tonometer (ICP; Icare Finland Oy, Helsinki, Finland). A previous version of the ICP was only able to measure IOP in the sitting position but with the pro version the clinician can measure IOP in both sitting and reclining positions.

TPA uses the same physical principle as GAT to measure IOP but the applanated area is much smaller (approximately 1.0mm in diameter) [9-12]. Ten consecutive readings are averaged and the result is provided along with a statistical confidence indicator.

ICP uses an impact rebound technique [13]. A small probe is accelerated against the cornea and the rebound acceleration is measured and translated into IOP [14-16]. The contact with the corneal surface is very brief and therefore no local anaesthesia is needed. This is an advantage in examination of children. Another advantage is that the rebound technique does not require continuous calibration. ICP averages six consecutive IOP measurements and provides the mean IOP out of these six measurements. ICP indicates the reliability of the measurements by a color code displayed below the IOP result. If the variation is within normal limits the indicator is green, yellow when the variation is greater than normal and the measurements should be viewed with caution and red when the variation is unacceptably high.

Whenever a device other than the gold standard is to be used, it should be one that offers increased precision relative to the gold standard. The aims of this study were to check if the repeatability of the ICP and TPA are comparable to GAT, if the IOP reading is equal in all three devices, and if there are any differences between IOP measured by GAT and by the two tested hand-held devices in sitting and reclining positions.

## Methods

36 eyes of 36 healthy volunteers (13 male and 26 females, 17 right and 19 left eyes) with a mean age of 41.9±13.8 years were included into this prospective, randomized, cross-sectional study. The study was performed at a single site, UniversityHospital Zurich, Switzerland, between November and December 2011. The study was pre-approved by the local Ethics committee (Cantonal Ethics Committee Zurich, Department of Health Canton Zurich, Zurich, Switzerland) and was conducted adhering to the tenets of the Helsinki Declaration and in compliance with all local and national regulations and directives. The study was registered to the Clinical Trials Register of the US National Institute of Health http://www.clinicaltrials.gov NCT01325324. Signed informed consent was obtained prior to the first examination.

Inclusion criteria were healthy ophthalmological status and age equal to or greater than 18 years. Exclusion criteria were a history of glaucoma or other ocular disease, and the presence of any corneal opacity or scarring that could affect IOP measurement. Only one eye of each individual was included. A randomization plan for choosing the eye to be enrolled in the study (right or left), and to determine the order in which the three tested tonometers were employed, and to minimize bias was generated by using http://www.randomization.com. Sample size calculations were done using a online tool of the Biostatistics Center of Massachusetts General Hospital, Harvard Medical School, Boston, Massachusetts, USA (http://hedwig.mgh.harvard.edu/sample_size/size.html). A total of 36 patients have entered this study. With a probability of 80 percent this study will detect a treatment difference at a two-sided 0.05 significance level, if the true difference between treatments is 2.4 mmHg based on standard deviation of 3.5 (or 1.8 mmHg based on a standard deviation of 2.7). IOP was measured first in an upright position with ICP, TPA and GAT (twice per device). Then, the patient reclined horizontally for 10 minutes and IOP was again measured twice with each of the hand-held devices (ICP and TPA). Between repeated measurement there was a pause of 3 minutes to avoid a lower IOP of the subsequent measurement caused by the prior applanation. If the statistical confidence indicator of the TPA was below 95% the measurement was repeated. ICP measurement was repeated until the quality indicator showed good repeatability (green). To minimize the influence of diurnal IOP fluctuation, all measurements were taken between 3 pm and 6 pm. Statistical analysis was performed using Excel for Windows (Microsoft Office 2003, Microsoft Corp., WA, USA) and SPSS/PASW statistics software (Version 18.0.0 for Macintosh, SPSS Inc. Chicago, IL, USA). The measurements were not normally distributed, as shown by Kolmogorov-Smirnov and Shapiro-Wilks tests. There was no post-hoc test with non-parametric Kuskall-Wallis-testing. Therefore, a logarithmic variable stabilizing transformation was performed to make use of one-way ANOVA. STATA™ (Version 10.1, StataCorp, Texas, USA) was used for the computation of the linear mixed models and Bland-Altman plots were created using MedCalc™ (MedCalc Software 7.3.0.1, Mariakerke, Belgium). Following the statistical analysis, differences between IOP values were considered statistically significant when p-values were less than 0.05.

For statistical analysis, mean IOP for each experimental condition for each of the three tonometers was calculated from two measurements. Coefficients of variation (COV) were determined for each tonometer and for sitting and reclining positions separately. Intraclass correlation coefficients (ICC) were determined with the procedure ‘xtmixed’ in STATA to evaluate differences in IOP between each of the three tonometers [17]. The linear mixed effects model was also used to evaluate differences in IOP measurement between sitting and reclining positions within ICP and TPA, respectively. In addition 95%-limits of agreement for consistency and the bias between the tonometers were evaluated by means of Bland-Altman analysis [18,19]. Limits of agreement are defined as the mean of the differences plus/minus 1.96 standard deviations (SD) of the differences. They provide an interval within which 95% of the differences between measurements by the two devices are expected to lie. The 95% confidence interval (95% CI) for the differences gives the additional information about the deterministic bias between both devices. If zero does not fall within the 95% CI we have to conclude that one of the methods measures deterministically higher values than the other.

## Results

Mean IOP measured by GAT (sitting) was 14.9±3.5 mmHg. Mean IOP measured by TPA was 14.8±2.7 mmHg whilst sitting upright and 17.0±3.0 mmHg in the reclining position. Mean IOP measured by ICP was 15.6±3.1 mmHg whilst sitting upright and 16.5±3.5 mmHg in the reclining position.

Coefficients of variation (COV) ranged from 2.9% for GAT to 6.9% for ICP in the reclining position. The COV can be found in Table 1. COV was best for GAT (2.9%). A statistically significant difference in COV was only found between ICP whilst reclining and GAT whilst sitting upright (p = 0.026).

**Table 1:** Coefficients of variation (COV)

	**COV Mean**	**SD**	**95%-CI**		**Median**	**Min**	**Max**	**p-value**
			**Lower**	**Upper**				
GAT (S)	0.029	0.034	0.018	0.041	0.000	0.000	0.109	-
ICP (S)	0.052	0.052	0.035	0.070	0.036	0.000	0.229	0.430
ICP (R)	0.069	0.059	0.049	0.089	0.050	0.000	0.224	0.026
TPA (S)	0.052	0.054	0.034	0.070	0.049	0.000	0.223	0.426
TPA (R)	0.042	0.048	0.035	0.070	0.036	0.000	0.229	0.878

(*GAT* Goldmann-Applanation-Tonometer, *ICP* Icare PRO, *TPA* Tono-Pen AVIA, *SD* Standard deviation, *CI* confidence interval, *S* sitting, *R* reclining).

The results obtained from the linear mixed model for the ICCs are provided in Table 2. ICCs ranged from 0.819 for ICP whilst reclining to 0.972 for GAT whilst sitting upright. The model was used to evaluate differences in IOP (ΔIOP) between GAT and all other devices (Table 2). IOP was higher measured by ICP and TPA compared to GAT with ΔIOP=0.847 mmHg for ICP in upright (p = 0.007) and ΔIOP=1.651 mmHg for ICP in reclining positions (p < 0.001), and ΔIOP=0.528 mmHg for TPA in sitting (p = 0.095) and ΔIOP=2.306 mmHg for TPA in reclining position (p < 0.001). With ICP, ΔIOP in the reclining compared to the upright position was −0.804 mmHg (SD = 0.297, 95%-CI −1.387, -0.221, p = 0.007). With TPA, ΔIOP in the reclining compared to the upright position was −1.778 mmHg (SD = 0.266, 95%-CI −2.300, -1.256, p < 0.001). In the reclining position ΔIOP between ICP compared to TPA was 0.654 mmHg (SD = 0.300, 95%-CI 0.066, 1.242, p = 0.029) whereas no significant difference could be found between both devices in the sitting upright position, with ΔIOP between ICP and TPA being −0.319 (SD = 0.295, 95%-CI −0.898, 0.259, p = 0.279).

**Table 2:** Results from the linear mixed model (ICC and ΔIOP)

	**ICC**	**SD**	**95%-CI**		**ΔIOP**	**SD**	**95%-CI**		**p-value**
			**Lower**	**Upper**			**Lower**	**Upper**	
GAT (S)	0.972	0.009	0.949	0.986	-	-	-	-	-
ICP (S)	0.866	0.042	0.767	0.931	0.847	0.316	0.228	1.466	0.007
ICP (R)	0.819	0.055	0.693	0.907	1.651	0.316	1.032	1.270	<0.001
TPA (S)	0.876	0.022	0.784	0.937	0.528	0.316	−0.091	1.147	0.095
TPA (R)	0.931	0.039	0.877	0.965	2.306	0.316	1.687	2.925	<0.001

(*GAT* Goldmann-Applanation-Tonometer, *ICP* Icare PRO, *TPA* Tono-Pen AVIA, *SD* Standard deviation, *CI* confidence interval, *ΔIOP* difference op IOP compared to GAT in mmHg, *S* sitting, *R* reclining).

**Figure 1:**
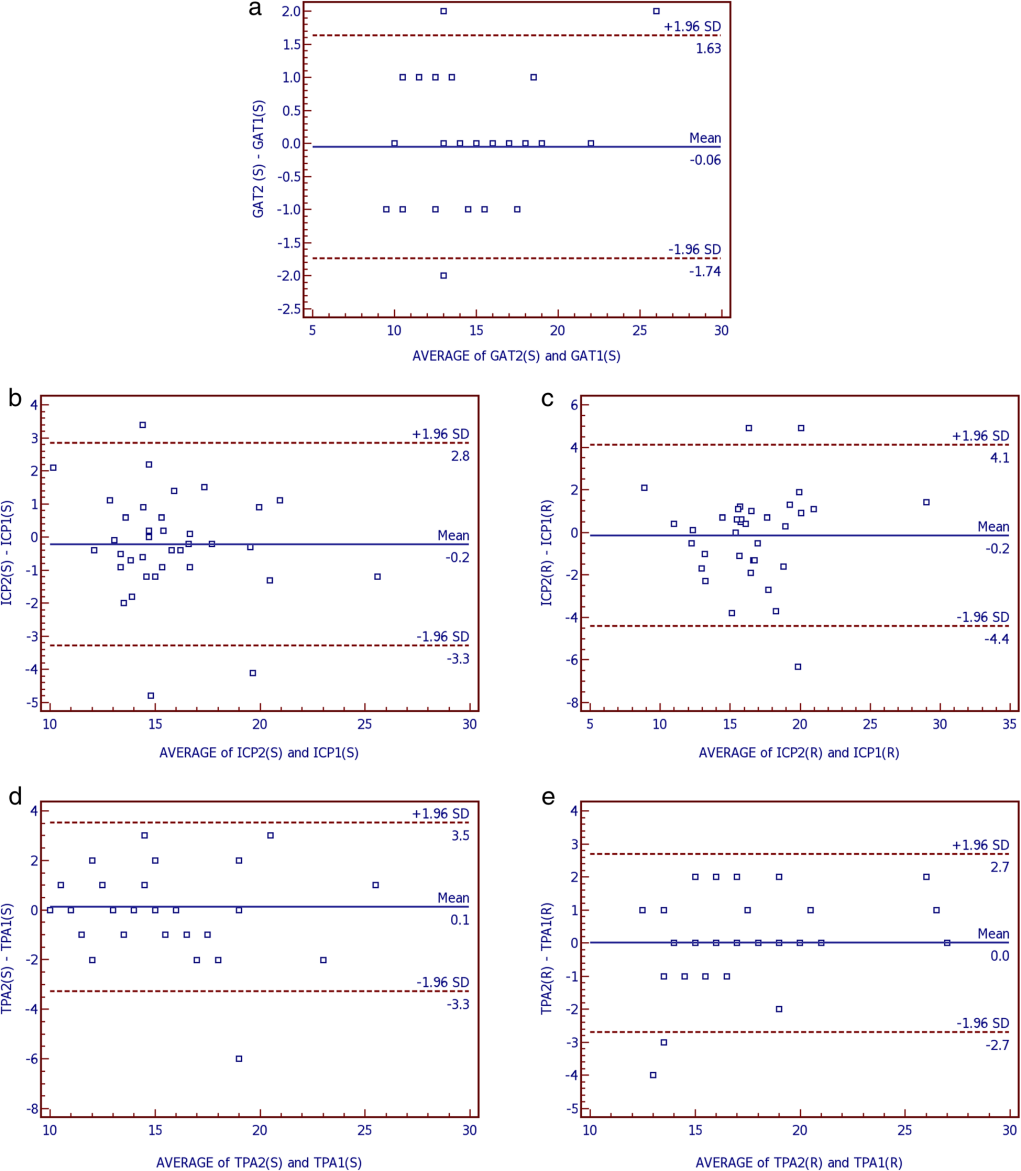
**Bland-Altman plots to demonstrate repeatability in measuring IOP (mmHg) between reading 1 and 2 with all devices (GAT a, ICP b and c and TPA d and e) and positions (sitting b and d and reclining c and e).** Limits of agreement were provided as 1.96-times standard deviation with upper and lower limit of the differences. Units for both axes are mmHg. (GAT = Goldmann-Applanation-Tonometer, ICP = Icare PRO, TPA = Tono-Pen AVIA, SD = Standard deviation, S = sitting, R = reclining).

**Figure 2:**
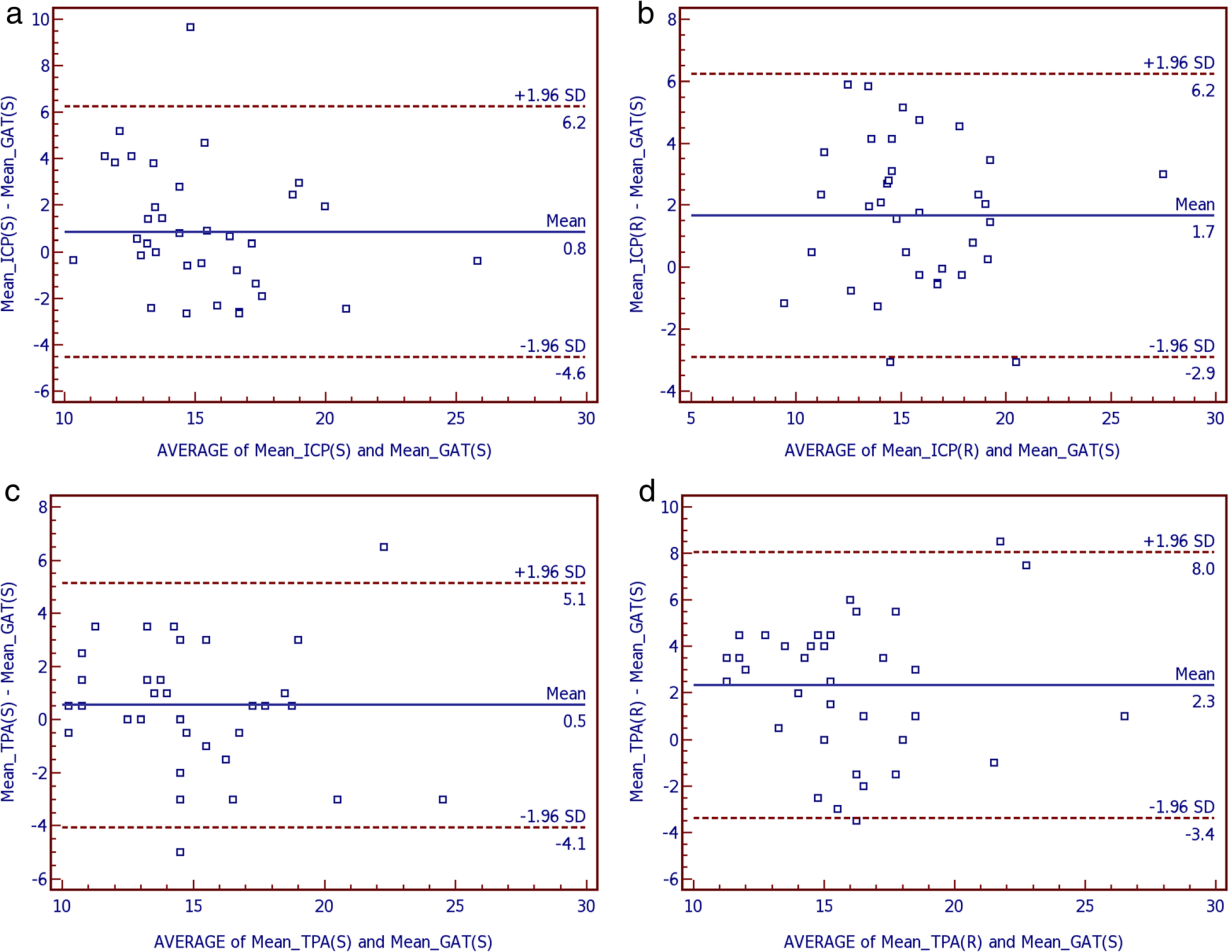
**Bland-Altman plots to demonstrate differences in mean IOP (mmHg) between Goldmann-Applanation-Tonometer and the two hand-held devices separately for both positions (sitting and reclining), Icare PRO (a, b) and Tono-Pen AVIA (c, d).** Limits of agreement were provided as 1.96-times standard deviation with upper and lower limit of the differences. Units for both axes are mmHg. (GAT = Goldmann-Applanation-Tonometer, ICP = Icare PRO, TPA = Tono-Pen AVIA, SD = Standard deviation, M = mean, S = sitting, R = reclining).

**Figure 3:**
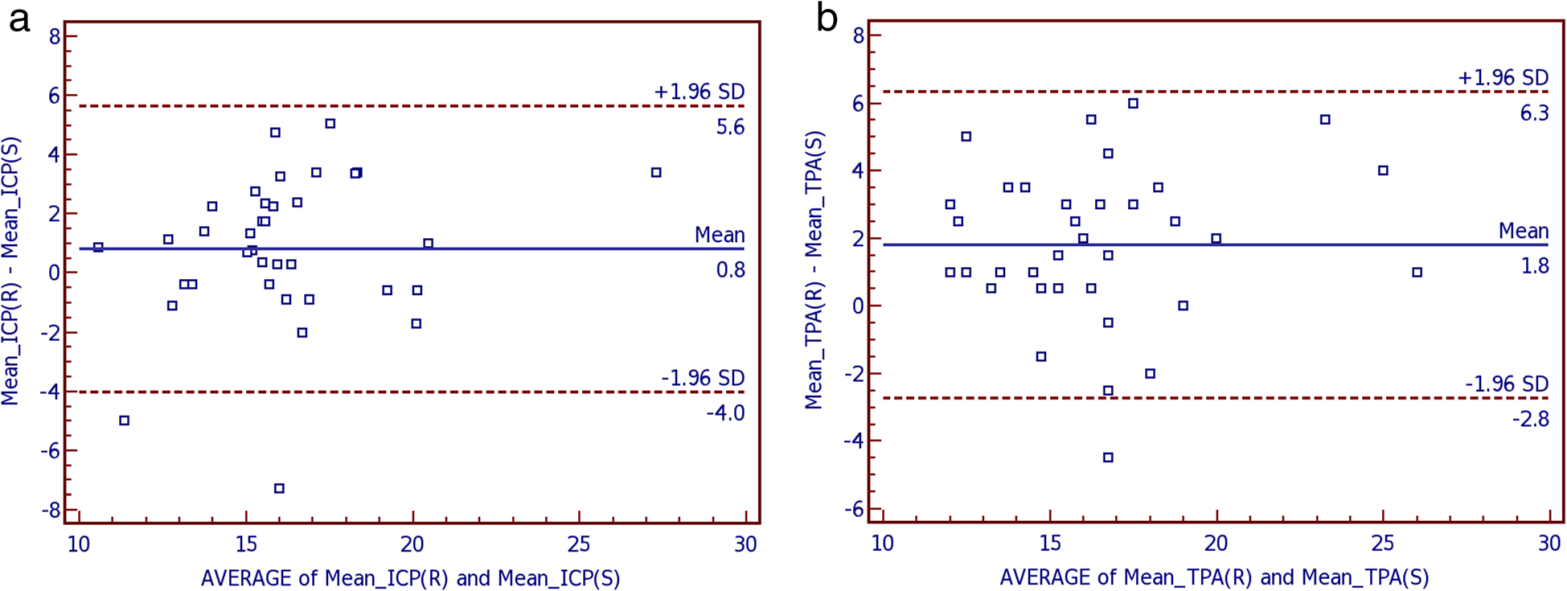
**Bland-Altman plots to demonstrate differences in IOP (mmHg) between sitting and reclining positions within the two hand-held tonometers, Icare PRO (a) and Tono-Pen AVIA (b).** Limits of agreement were provided as 1.96-times standard deviation with upper and lower limit of the differences. Units for both axes are mmHg. (ICP = Icare PRO, TPA = Tono-Pen AVIA, SD = Standard deviation, M = mean, S = sitting, R = reclining).

Bland-Altman plots were used to demonstrate differences between measurement 1 and 2 of the three methods in sitting position (Figure 1). Furthermore, Bland-Altman plots were used to show differences between both hand-held tonometers in both positions (sitting and reclining) compared to GAT (Figure 2) and between sitting and reclining positions within both hand-held tonometers (Figure 3). Bias and limits of agreement as well as confidence intervals and p-values are provided in Table 3. Bland Altman plots show good repeatability between IOP reading 1 and 2 (Figure 1). Bias was very low for all devices and ranges between 0.0 mmHg for TPA in reclining and 0.1 mmHg for TPA in upright positions. IOP was higher measured by ICP and TPA compared to GAT by 0.8 mmHg for ICP in sitting and 1.7 mmHg for ICP in reclining position, and by 0.5 mmHg for TPA in sitting and 2.3 mmHg for TPA in reclining positions (Figure 2). IOP was higher in the reclining compared with upright position (Figure 3). The effect was greater for TPA (1.8 mmHg) than for ICP (0.8 mmHg).

**Table 3:** Summaries of the Bland-Altman plots

	**Bias**	**Limits of agreement**		**95%-CI**		**p-value**
		**Lower**	**Upper**	**Lower**	**Upper**	
GAT2 (S) – GAT1 (S)	−0.06	−1.74	1.63	−0.235	0.347	0.701
ICP2 (S) – ICP1 (S)	−0.2	−3.3	2.8	−0.312	0.745	0.411
ICP2 (R) – ICP1 (R)	−0.2	−4.4	4.1	−0.584	0.890	0.676
TPA2 (S) – TPA1 (S)	0.1	−3.3	3.5	−0.699	0.477	0.703
TPA2 (R) – TPA1 (R)	0.0	−2.7	2.7	−0.465	0.465	1.000
MICP (S) – MGAT (S)	0.8	−4.6	6.2	−0.085	1.780	0.074
MICP (R) – MGAT (S)	1.7	−2.9	6.2	0.864	2.439	<0.001
MTPA (S) – MGAT (S)	0.5	−4.1	5.1	−0.266	1.321	0.1856
MTPA (R) – MGAT (S)	2.3	−3.4	8.0	1.319	3.292	< 0.001
MICP (R) – MICP (S)	0.8	−4.0	5.6	−0.032	1.640	0.059
MTPA (R) – MTPA (S)	1.8	−2.8	6.3	0.995	2.560	<0.001

The agreement between measurement 1 and 2 with each tonometer and between GAT and each hand-held tonometer and position (sitting and reclining) and between sitting and reclining positions within both hand-held tonometers was investigated. Lower and upper limits of agreement were provided as 1.96-times standard deviation of the differences (SD). Bias, 95% limits of agreement and 95% confidence interval are provided in mmHg.

(*GAT* Goldmann-Applanation-Tonometer, *ICP* Icare PRO, *TPA* Tono-Pen AVIA, *CI* confidence interval, *M* mean, *S* sitting, *R* reclining).

## Discussion

The accurate measurement of intraocular pressure is important in managing many ophthalmic diseases and conditions, e.g. glaucoma, uveitis, and traumatic conditions such as hyphema. The most accurate method of measuring intraocular pressure remains cannulation of anterior chamber and direct manometry. Because of its invasive nature and the risk of adverse effects, e.g. infection, this method is reserved for some experimental designs only. Hence, the gold standard of clinical IOP measurement remains Goldmann applanation tonometry. GAT can routinely be performed in the consultation room with a slit lamp-mounted device. However, GAT can only be performed whilst the patient is sitting upright at the slit lamp. In some instances, for example post-trauma examination in the emergency room or an intensive care unit, it is impossible to use the slit lamp-mounted GAT. In these conditions a hand-held device is used to measure IOP. Sometimes it is only possible to examine the patient whilst they are reclining horizontally. Whenever the use of a tonometer other than the gold standard GAT is considered, the operator should know if IOP measurements with the hand-held devices are reliable and by how much the IOP in these settings differs from IOP measurement with GAT in the upright position.

The aim of this study was to evaluate these two questions. Firstly, the repeatability of all devices was tested. Therefore, COVs and ICCs were calculated. COVs and ICCs were good for all three devices and both positions (sitting upright as well as reclining). Nevertheless, GAT showed the lowest COV and highest ICC. A significant difference for COVs could only be detected between GAT in sitting and ICP in reclining positions. ICC was best (highest) for GAT and worst (lowest) for ICP in the reclining position. In the latter case there was even no overlapping of the 95% confidence intervals. Bland-Altman analysis was used to check the test-retest repeatability of one single operator in the upright and reclining positions. Bland-Altman analysis shows good repeatability with low bias between test (measurement one) and retest (measurement 2) for all three tested devices and in both positions. Limits of agreement were best for GAT compared to the two hand-held devices.

Second, this study evaluated the amount in which the IOP measured by the hand-held devices in both upright and reclining positions differs from the IOP measurement with GAT in the upright position. Mean IOP differs between all tonometers, and between upright and reclining positions when using ICP or TPA. IOP was generally lower in the sitting upright compared to the reclining position. This is consistent with previous studies [20-22]. The effect was greater for the TPA than for the ICP. This has been evaluated in other studies with a previous model of the used Icare PRO, named Icare. But the Icare was only able to measure IOP in the upright position, and not whilst the patient is reclining. To our knowledge there are no prior studies comparing GAT and TPA with the new Icare PRO.

IOP measurements are dependent on central corneal thickness (CCT). Limitations of our study are that we do not correlate the measurements with CCT and only healthy individuals are included. In healthy individuals we do not expect an unacceptably large range of CCT. Furthermore, it is not standard clinical practice to measure CCT in a healthy patient. Therefore, in a standard clinical setting the hand-held tonometers will be used without knowing the CCT of these eyes, which could lead to an unknown bias. However, there is a lack of consensus on the influence of corneal thickness and axial length on IOP measurements. Regarding rebound tonometry, one study found no correlation between CCT and IOP [23], while others found a correlation [24,25]. It is known that accuracy of IOP measurement is affected in eyes with corneal pathologies, e.g. post keratoplasty, with corneal scarring, with high or irregular astigmatism, or in the presence of corneal edema [26-31]. This study did not check repeatability in eyes with corneal pathologies. Another limitation of this study is that only eyes with IOP between 9 and 27 mmHg (GAT) were included, which reflects normal and slightly elevated IOP. It is known that IOP measured by Tono-Pen corresponds well with GAT in the range 9-20 mmHg, but underestimates IOP ≥30 mmHg and overestimates IOP ≤9 mmHg [32-34]. Further studies should evaluate ICP in a larger sample size including eyes with elevated IOP and a matched group of patients with glaucoma.

## Conclusion

In a clinical setting all three devices may be used because of their good repeatability. But regarding repeatability, COV and ICC were superior only for GAT, the other devices should only be used when it is not possible to use a slit-lamp mounted GAT. TPA has been widely used for many years now. ICP is a recently-introduced alternative to TPA. Because no local anaesthesia is needed, ICP is a good way to measure IOP especially in children or in adults who are unable to fully co-operate. If it is impossible to measure in the upright position one can use TPA or ICP. IOP measurements between GAT, TPA and ICP as well as between upright and reclining positions are not interchangeable. If decisions depend on the exact value of IOP, there is the desire to use conversion factors to compare IOP readings with GAT. Nevertheless, at the moment none conversion formula exists that would be accurately applicable.

## Competing interests

The authors report no conflicts of interest. The authors alone are responsible for the content and writing of the paper. No financial support was received.

## Authors’ contributors

MT-H and JF designed the study, monitored data collection, and conducted the statistical analysis, and interpretation of data. CS conducted the study, and collected the data. MT-H wrote the initial draft of the paper. JF, CS and JVMH contributed to revision of the paper. All authors read and approved the final manuscript.

## Pre-publication history

The pre-publication history for this paper can be accessed here:

http://www.biomedcentral.com/1471-2415/13/44/prepub
